# Mindfulness at Methodist—A Prospective Pilot Study of Mindfulness and Stress Resiliency Interventions in Patients at a Tertiary Care Medical Center

**DOI:** 10.3390/ijerph18084034

**Published:** 2021-04-12

**Authors:** Elaina Vivian, Hellen Oduor, Preeti Girisha, Parvez Mantry

**Affiliations:** 1 Methodist Digestive Institute, Methodist Dallas Medical Center, Dallas, TX 75203, USA; ElainaVivian@mhd.com; 2 The Transplant Institute, Methodist Dallas Medical Center, Dallas, TX 75203, USA; HellenOduor@mhd.com; 3 True Self Therapy, S-Corp, Frisco, TX 75036, USA; preeti@trueselftherapy.us; 4 The Liver Institute, Methodist Dallas Medical Center, Dallas, TX 75203, USA; 5 Clinical Research Institute, Methodist Health System, Dallas, TX 75203, USA

**Keywords:** mindfulness, perceived stress, stress reduction, oncology

## Abstract

Individuals with chronic medical conditions like cancer often experience heightened stress levels that can impact medical decision-making. The aim of this study was assess the impact of mindful stress-reduction interventions in cancer patients and support group participants (which included current and former cancer patients and their caregivers). A pilot study was conducted in which participants were provided a mindful stress-reduction intervention to determine whether they reduced stress, anxiety, and communication issues. Participants were provided a one-hour mindful stress-reduction intervention by a licensed physical therapist. Surveys were given to participants immediately before and after, and again 7-days after the intervention. Perceived stress was ascertained by asking participants: “Which emotional/mental state do you most frequently find yourself in?” Anxiety and communication abilities were measured using Neuro-QoL™ Anxiety and Communication v.1 instruments. Fifty-nine participants with a mean age of 60.6 years completed the study. Of these, 30.5%, 6.8%, 23.7%, and 39% were diagnosed (or were a caregiver to someone diagnosed) with pancreas, liver, breast, or unknown cancers, respectively. The surveys showed that participants’ perceived stress scores (*p* < 0.001), anxiety levels (*p* = 0.0067), and pain scores (*p* < 0.0001) were reduced after the mindful stress-reduction intervention. Larger studies with control groups are needed to confirm the interventions’ benefits.

## 1. Background

Cancer represents the second leading causes of mortality in the United States. The National Center for Health Statistics reported that in 2019, there were data from the National Vital Statistics System indicating that there were over a half-million deaths attributable to cancer [1]. The American Cancer Society has determined that the most frequently diagnosed cancers in the US are breast, lung, and prostate cancer, representing a combined total of almost 700,000 new cases in 2020 [2]. Lung, prostate, breast, and pancreas cancers lead in mortality, with over 300,000 estimated 2020 deaths [2]. Treatment options are varied, and can include single or combination regimens of surgery, chemotherapy, immunotherapy, targeted therapy, hormone therapy, or stem cell transplants [3], All of these cancer treatment options can have physically, mentally, spiritually and financially taxing impacts on patients and their support networks [4].

Patients with a chronic medical condition like cancer not only experience the acute stress associated with the original diagnosis, but also chronic stress associated with ailment progression and their perception on how information about their treatment options is relayed to them [5]. A cancer diagnosis brings a degree of uncertainty that makes medical decisions uniquely stressful. Patients and their caregivers must weigh variables like provider recommendations, efficacy, side effects, and overall survival rates to decide on a course of treatment, all while contending with a heightened level of stress and emotional intensity [6]. Furthermore, hospitalized patients may experience elevated stress levels related to fear of the unknown, unfamiliar surroundings, and coping with hospitalization [7]. Support group participants represent patients and caregivers in various stages of their treatment journeys. These individuals may also experience heightened levels of stress and anxiety, perhaps dependent on where they are in their treatment continuum.

Individuals who have chronic episodes of stress are at an increased risk of impaired performance, reduced resilience, poor decision-making, exhaustion, and reduced immune competence [8,9,10]. For both patients and caregivers, stress management resources are inefficient and/or not readily recognizable or available [11].

Mindfulness-based stress reduction (MBSR) therapy has been shown to be a powerful tool for patients dealing with chronic conditions [12]. MBSR therapies help those involved to control their perceptions of current events and to respond appropriately by empowering participants to control both internal and external stress factors [13]. MBSR therapies provide several benefits for participants, including stress reduction, emotion regulation [14,15], increased working memory [16], higher levels of self-satisfaction [17], improved focus [18], and reduced emotional exhaustion [19]. MBSR is an eight-week structured program delivered by certified trainers that includes group meetings, one-day retreats, and homework, and instruction in mindfulness meditation, body scanning and simply yoga postures [20]. Despite its demonstrated advantages; the time required for undergoing MBSR training makes it difficult to replicate for some patient populations.

A recent survey of cancer patients and caregivers about their decision-making preferences and the impact of stress on their treatment journey showed that the majority of patients felt stress adversely affected communication between themselves and their care providers [11]. In addition, patients thought it pertinent that the hospital provide resources to help manage their stress, but few reported receiving any stress management resources. The Advisory Board conducted a cancer patient experience survey in 2019 and similarly found that 75% of patients (*n* = 1201) reported that relaxation therapies would have been valuable during their experience with cancer [21].

Implementation of tailored mindful stress-reduction interventions in selective populations would provide immediate information involving application success and opportunities for improvement. The aim of this study was to assess the effectiveness of mindful stress-reduction interventions, which were based on some of the premises found in MBSR but delivered as a one-time experience, on alleviating stress, anxiety, and pain levels, while improving communication abilities primarily in cancer patients and support group participants at a tertiary care medical center. A secondary aim was to investigate the degree of agreement between outpatients and their provider regarding shared decision-making activities during a pivotal treatment-planning visit. As a pilot study, we also sought to evaluate the feasibility of such an intervention in our patient populations.

## 2. Methods

### 2.1. Study Objectives and Outcomes

The primary objective of the pilot study was to assess the effectiveness of mindful stress-reduction interventions on participants stress, anxiety, communication, and pain levels (for some patients) at a tertiary care medical center. Participants fell into three different natural groups, which included:

⧫ Cancer inpatients who received the intervention in a one-on-one fashion
⧫ Cancer outpatients who received the intervention in a one-on-one fashion
⧫ Support group participants (i.e., current or former cancer patients and their caregivers) who received the intervention in a group setting

The primary objective was assessed by administering questionnaires immediately before and after the intervention, and again 7-days after the intervention to measure participants’ self-reported stress, anxiety, and communication abilities. Pain was only accessed for cancer inpatients immediately before and after the intervention. See Table 1 for timeline of when participants received questionnaires.

A secondary objective was to determine if there was agreement between outpatients and their physician’s perceptions regarding shared decision-making conversations during a pivotal treatment planning visits. The secondary objective was assessed by administering a questionnaire immediately before the intervention and immediately after the treatment appointment to determine levels of agreement between patients and their provider. See Table 1 for timeline of when participants received questionnaires.

### 2.2. Feasibility of the Pilot Study Was Ascertained by Intervention Completion and Survey Response Rates. Study Setting and Participants

This prospective single-center interventional pilot study was carried out at Methodist Dallas Medical Center (MDMC) between 1 June 2018 and 30 June 2019. IRB review and approval were obtained from Aspire Independent Review Board (Protocol 030.HEP.2018.D). The trial was retrospectively registered on ClinicalTrials.gov under the trial record number NCT04255082 on 5 February 2020. The URL of the trial registry is: https://clinicaltrials.gov/ct2/show/NCT04255082?term=NCT04255082&draw=2&rank=1. Informed consent was obtained from all individual participants included in the study.

**Figure 1:**
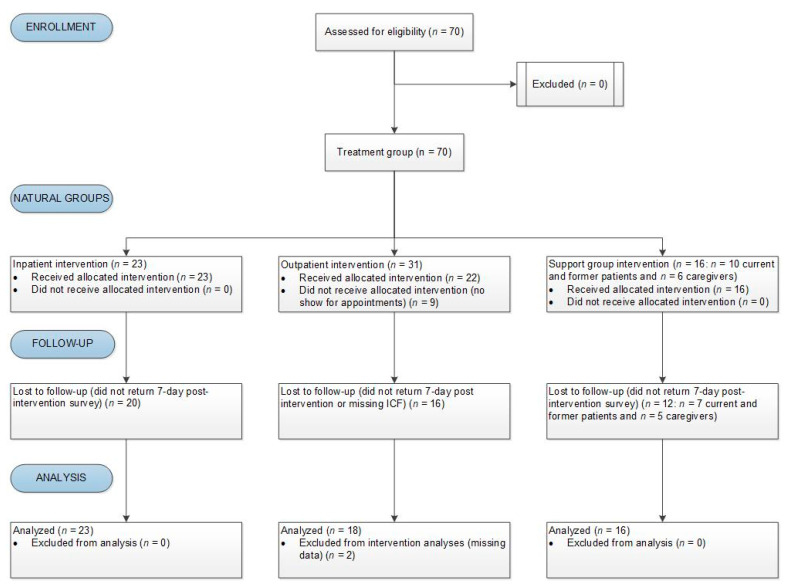
CONSORT patient stratification diagram.

The study inclusion criteria specified that participants had to be 18 years old or older; and were currently or formerly receiving cancer treatment or were a caregiver to someone receiving cancer treatment. Participants were either inpatients at MDMC; outpatients receiving services from MDMC or the Liver Institute at MDMC; or participants of MDMC’s Breast or Pancreas Cancer Support Groups (which included current or former patients and their caregivers) (see Figure 1 for Consolidated Standards of Reporting Trials (CONSORT) patient stratification diagram)**.** Inpatients were identified by their current bedside nurses at medical/surgical and oncology units’ Interdisciplinary Plan of Care meetings. These participants were identified as good study candidates after providing them a description of the study and aims, and completing informal verbal assessments of patients’ willingness, mood, pain levels, and clinical disposition. Inpatients underwent the intervention with the licensed physical therapist for one hour.

Outpatients were identified and scheduled to participate in the study intervention by the patients’ designated nurse navigator one hour before the patients’ treatment plan appointment with their physician or treatment planning team. Clinicians were also asked to complete shared decision-making surveys after treatment planning appointments with outpatients.

Support group participants (current and former patients and caregivers) underwent the intervention with the licensed physical therapist for one hour as a group. All participants provided written informed consent.

Study completion was defined as a participant completing the intervention, and only the immediate pre- and post-intervention questionnaires.

### 2.3. Mindful Stress-Reduction Interventions

A licensed physical therapist certified in professional medical therapeutic yoga (Professional Yoga Therapy Institute^®^ certified (PYTI-C)), as well as having significant experience with mindfulness and stress reduction techniques, was contracted to develop and provide all study interventions to participants. These interventions included:

- Creating a therapeutic landscape to open communication with participants [22]. Allowing landscape helps to establish a trusted relationship between practitioner and person fostering openness and willingness to change [23].
- Postural (kinesthetic) awareness through guided imagery (somatosensory integration), which is the sense of placement in space and sense of size, shape and texture of things on the bodies surface.
- Postural stability, which is the focus of stability to create safety for a movement. In a stable pose the central nervous system is quieted as fewer signals are sent making external and internal relaxation easier [24].
- Breathing exercises/voluntary breathing, breathing with conscious control that requires focus [25].
  ○ Abdomino-diaphragmatic breath [26] is at rest inhalation with a passive descent of the respiratory diaphragm, which is critical to normal autonomic function and stress regulation [27]. This works through induction of inhibitory nerve impulses to baroreceptors in the carotid that monitor blood pressure and heart rate. The diaphragmatic descent stimulates slow adapting stretch receptors to down regulate sympathetic tone and the hypothalamic-pituitary-adrenal axis.
  ○ Coherence breathing [28] works to elongate breath, equalizing inhale and exhale breaths to achieve optimal respiratory rate for improving heart rate variability near 6 breaths/minute [29].

The physical therapist met with participants individually or in groups, obtained informed consent, delivered a one-hour study intervention, and administered and collected the pre- and post-survey instruments. A member of the research team mailed study participants a seven-day follow-up survey with a thank-you gift card.

### 2.4. Stress Intervention Assessments

#### 2.4.1. Perceived Stress

Perceived stress, also described as brain/emotional state [30], was ascertained by asking respondents “Which emotional/mental state do you most frequently find yourself in?” pre- and post-intervention. Previous studies showed that responses to the perceived stress question were significantly associated with their self-reported frequency of work-related stressors [6,31], demonstrating some face validity. Responses to the perceived stress question were rated on a five-point Likert scale: 1 = feeling great! 2 = feeling good, 3 = a little stressed, 4 = definitely stressed, and 5 = stressed out.

#### 2.4.2. Anxiety

A patient-reported outcome (PRO) measurement tool, the Neuro-QoL™ Item Bank v1.0 –Anxiety instrument (HealthMeasures, Northwestern University; www.healthmeasures.net, accessed on 03 April 2018), was used to assess participants’ pre-intervention and seven-day post-intervention anxiety levels. The instrument has been thoroughly tested for reliability and is available for public use (Cronbach α = 0.95) [32]. The Neuro-QoL™ Item Bank v1.0 –Anxiety instrument contains 21 statements about the frequency in which participants experience anxiety symptoms in the past seven days. The statements are answered using a five-point Likert scale (i.e., 1 = never to 5 = always) [33]. A raw score is calculated by summing the scores from all of the responses. The range of possible raw scores is 21 to 105, with higher scores indicating worse (undesirable) self-reported anxiety.

#### 2.4.3. Communication

Another PRO tool, The Neuro-QoL™ Scale v1.0—Communication instrument (HealthMeasures, Northwestern University; www.healthmeasures.net, accessed on 3 April 2018), was used to assess participants’ pre-intervention and seven-day post-intervention communication abilities. Neuro-QoL™ instruments are supported by substantial quality and quantitative evidence that supports validity [34]. However, to our knowledge this data is not available for the individual Neuro-QoL™ Communication scale. The Neuro-QoL™ Scale v1.0—Communication short form instrument contains five statements about the frequency in which participants experienced communication difficulties in the past seven days. The statements are answered on a five-point Likert scale (i.e., 5 = none to 1 = cannot do). Scores are derived by summing the values of each answer, then subtracting five from the total sum, multiplying by 100 and dividing by 20. The range of possible scores is 0 to 100. Higher scores indicate better (desirable) patient-reported communication abilities [35].

#### 2.4.4. Visual Analog Scale (VAS) Pain Score

The pain VAS is a one-dimensional measure of pain intensity and is a highly subjective pain assessment tool for clinical settings [36], which has been shown to have high internal consistency (Cronbach’s α = 0.91) [37]. The VAS pain scores range from 0 = no pain, 5 = moderate pain, and 10 = worst pain. Only inpatients were asked to indicate pain intensity level using the pain VAS.

#### 2.4.5. Shared Decision-Making

The Dyadic OPTION (observing patient involvement) instrument was used to assess shared-decision making from both the patient and clinician viewpoint. At the time of this study, there was no reliability or validity data yet available. The Dyadic OPTION instrument contains 12 statements about how elements of shared decision-making were perceived during the appointment [38]. Answers to the statements were given on a Likert-type scale (i.e., strongly agree, agree, disagree and strongly disagree). Only outpatient study participants and their physicians were asked to complete identical surveys on being, or feeling, involved in shared decision-making during an appointment immediately following the mindful stress-reduction intervention.

### 2.5. Statistical Analysis

Descriptive statistics are reported as absolute frequencies (*n*), mean  ±  standard deviation, and median (range) for continuous variables. Categorical variables are reported as absolute (*n*) and relative frequencies (%). Continuous variables included pre- and post-intervention anxiety raw score, communication raw score, and VAS pain score, which were evaluated for normality using the graphical normal probability plot (i.e., QQ plot); pre- and post-differences were evaluated using paired *t*-tests. Relational group concepts were evaluated by calculating Pearson’s correlation coefficients between pre- and post-intervention anxiety raw scores, communication raw scores, perceived stress, and VAS pain scores. Parametric analysis of variance (ANOVA) tests were used to evaluate group differences between pre- and post-intervention perceived stress and anxiety, communication, and VAS pain scores. Significance was defined as *p*  <  0.05. All analyses were conducted using Statistical Analysis System (SAS) v. 9.4 (SAS Institute Inc., Cary, NC, USA).

## 3. Results

Between 1 June 2018 and 30 June 2019, 70 participants were recruited and signed-up for study visits. Of these, 59 (84.3%) completed the study (Figure 1). Fourteen participants (27.1%) who completed the study returned the seven-day post-intervention follow-up surveys. Participants were outpatients (33.9%), inpatients (39.0%), and those who attended MDMC support groups (27.1%). They were 60.7% female and58.9% White. On average, participants were 60.6 (±14.6) years old and most had pancreas (30.5%), liver (6.8%), and breast (23.7%) cancer diagnoses. Thirty-nine percent of participants had or were caregivers to someone with other/unknown malignancies that were not disclosed on the returned study surveys (Table 2).

### 3.1. Intervention-Associated Outcomes

To assess perceived stress, participants were queried on how they would rate the emotional/mental state that they were most frequently in before and after the mindful stress-reduction intervention. Before intervention, 73.7% of participants reported either feeling a little stressed, definitely stressed, or stressed out (Table 3).

After the intervention, participants’ perceived stress levels were reduced when compared to baseline whereby 18.5% participants reported feeling a little stressed and none reported feeling definitely stressed or stressed out. Among outpatients assessed following their treatment planning appointment with their physician, 18.2% reported feeling a little stressed and none reported feeling definitely stressed or stressed out. Analysis of anxiety levels revealed that participants’ seven-day post-intervention scores were significantly lower than their pre-intervention anxiety raw scores (*p* = 0.0067) (Table 4). Comparison of communication difficulties pre-intervention and seven days post-intervention showed that pre-intervention raw scores were significantly lower than post-intervention communication raw scores (*p* = 0.0135) indicating improved communication abilities after mindful stress-reduction interventions. Twenty-one inpatients reported having pain greater than 0 on the VAS before mindful stress-reduction interventions. In addition, the average pre-intervention pain score was significantly higher than post-intervention scores (*p* < 0.0001).

### 3.2. Intervention-Associated Outcomes by Demographics

Intervention-associated outcomes by demographics refer to all the participants who completed the intervention and the questionnaires administered immediately before and after that intervention (*n* = 59). There were no differences in perceived stress, pain, communication abilities in pre- or post-interventions by gender, race, ethnicity, or age. However, pre-intervention anxiety scores were significantly higher in women than men (*p* = 0.0063). This pattern held for post-intervention anxiety scores in women and men (*p* = 0.0179). Younger participants aged 25 to 44 had lower anxiety raw scores compared to those aged 45 to 64, (*p* = 0.0375) (data not shown).

No differences in perceived stress, anxiety, or communication abilities in pre- or post-interventions by diagnosis were found. However, pre-intervention pain scores were highest for inpatients with unknown/other malignancies and pancreas cancer, compared to breast cancer patients, (*p* = 0.0008) (data not shown).

There were also no differences between types of participants (i.e., inpatient, outpatient, and support group participants) and pre- and post-intervention perceived stress or communication abilities. However, support group participants reported the least post-intervention perceived stress (75% reported feeling great!) compared to inpatients (19.0%) and outpatients (11.8%) (*p* = 0.0003). Although not statistically significant, inpatients had higher pre-intervention anxiety compared to outpatients (*p* = 0.0505) (data not shown).

### 3.3. Relationships Between Anxiety, Stress, Communication and Pain

A significant relationship was observed between pre-intervention perceived stress levels and pre-intervention anxiety raw scores (*p* = 0.0195; Table 5). The group with the highest perceived stress level (stressed out! = 5) reported the highest anxiety raw scores, whereas the group with the lowest perceived stress level (feeling great! = 1) reported the lowest anxiety raw scores.

A negative correlation was observed between pre-intervention anxiety raw scores and pre-intervention communication raw scores (*p* = 0.0002), indicating that as anxiety increased, communication abilities were negatively affected (Table 6). The same pattern was true for post-intervention anxiety raw scores and post-intervention communication (*p* = 0.0329). Although not statistically significant, correlation analyses found that pre-intervention anxiety raw scores were weakly (and positively) associated with pre-intervention VAS pain scores (*p* = 0.1506). This suggests that as anxiety increases, physical manifestations of pain also increase.

### 3.4. Shared Decision-Making

Changes in shared decision-making between pre- and post-mindful stress-reduction interventions were assessed using the Dyadic OPTION Scale for Shared Decision-Making [38]. As the Dyadic OPTION Scale is not a formally validated instrument [39], only response frequencies are presented. Eleven outpatients and eight physicians completed these surveys after a treatment planning appointment (Table 7). For all items on the survey, 72.7% of outpatients agreed or strongly agreed with the statements and 100% of physicians agreed or strongly agreed with all statements. The greatest discrepancy between outpatient and physician responses was observed with the item, “Concerns or worries about managing the health problem were discussed.” Among the participants, 27.3% disagreed or strongly disagreed that this had occurred, while 100% of physicians agreed or strongly agreed with the statement.

## 4. Discussion

The present study explored the short-term effects of mindful stress-reduction interventions on cancer patients and caregivers. Our findings show that the intervention resulted in reduced anxiety, pain scores, and perceived stress levels with concomitant increased ease of communication among participants. The intervention was not intended to replace traditional medical therapy, but rather, as a complement. This is in line with the current practice of the use of MBSR interventions in the field of psycho-oncology to train individuals to use their innate resources and abilities to respond more effectively to stress, pain, and illness.

### 4.1. Review of Findings

The results demonstrate that a mindful stress-reduction interventions can, in the short term, effectively: (1) reduce participants anxiety raw scores, (2) reduce participants’ self-reported perceived stress levels, (3) decrease participants’ self-reported difficulty in communication, and (4) decrease pain scores among hospitalized patients. Further, analysis demonstrated (5) non-congruence in the perception of whether “concerns or worries about managing the health problem were discussed” among outpatients and their providers; providers unanimously perceived that this had occurred, while more than a quarter of the outpatients perceived that this was not the case. This particular finding suggests the need for a more deliberate discussion on coping skills, especially during pivotal office visits, and clearer communication between both parties. Finally, the results demonstrate (6) feasibility of such an intervention in inpatient, outpatient, and support group settings as shown by enrollment of eligible participants, 84.3% study completion rate, and 23.7% seven-day post-intervention survey response rate.

The original impetus for this study was findings from our own institutional study. That study found that the majority of patients acknowledged that stress adversely affected their treatment plans and interactions with their care team, but they did not receive any stress management information or interventions, despite their preference to receive them [11]. This study contributes to the foundation of future research in this area by demonstrating feasibility and successful short-term results of such an intervention.

Although our intervention was significantly different and shorter, our interventions’ findings are consistent with those of Hoffman [40] and Bränström [41] which found that MBSR interventions had a positive effect on stress and anxiety. Further, patients contending with chronic illnesses identified numerous points of stress that originated from their medical condition as well as from how information relayed to them about their treatment options made them feel. MBSR therapies help those involved to control their perceptions of current events and to respond appropriately to them [13]. These therapies empower participants to control both internal and external stress factors. This increased control and awareness makes MBSR a powerful tool for medical patients who deal with chronic conditions [12]. Medical patients with anxiety disorders have also been found to benefit from MBSR [42,43]. This demonstrates the ability of the process to transcend the role of doctor and patient, and benefit anyone engaged in it.

We found that women had higher anxiety levels compared to men, which is consistent with previously published research [44,45,46]. Bahrami and Yosefi demonstrated that this disparity may be attributable to women being more likely to believe in the uncontrollability of worry, thereby suffering from more social anxiety, health anxiety, and metaworry (worry about worry) than men [44].

One unique aspect of our study design was the assessment of shared decision-making perception between outpatients and providers. The hypothesis was that outpatients could be better prepared to participate in shared decision-making following mindful stress-reduction interventions due to having a more relaxed disposition. We posit that the incongruence in provider versus patient perception of whether “concerns or worries about managing the health problem were discussed” may be a function of experimenter effects and social desirability bias. It is possible that the providers gave the desired response to appear more favorable to the researchers. An alternative explanation could be that completing the survey may have been viewed by providers as an inconvenience in addition to an already demanding outpatient clinic schedule; leading them to select the same response (“Agree/Strongly Agree”) to all survey questions.

### 4.2. Limitations and Future Research

Although the findings from this study are significant and consistent with the growing body of evidence, there are some limitations and suggestions for future research. Since it was outside the scope of this study to analyze the long-term effects of the intervention, it cannot be concluded that the short-term results from one mindful stress-reduction intervention session will be sustained and/or useful in helping participants cope with the ongoing stress associated with a cancer diagnosis. We suggest interventions that go beyond one session and additional research is warranted to determine how long the beneficial effects of interventions like ours persist.

Support group participants reported having the least amount of post-intervention perceived stress, which may indicate differences in the way individual and group sessions were performed. Patients actively undergoing acute or ongoing medical treatment may be more anxious and/or stressed [47,48]. Our study showed that inpatients had higher pre-intervention anxiety compared to outpatients, which further suggests setting differences in pre- and post-intervention outcomes.

It is well established that both patients and their caregivers experience stress and anxiety during the cancer treatment journey. We did not investigate differences between patients and their caregivers in this study, however, Li et al. found that adults with cancer and family caregivers reported similar degrees of anxiety and depression [49]. It has also been reported that caregivers may experience equal or greater degrees of psychological distress that adults with cancer [50,51].

Furthermore, there are many additional interfering factors which may impact an individual’s perceived stress and anxiety levels that were not taken into account in this study. The World Health Organization released a comprehensive report which comprehensively described how “mental health and many common mental disorders are shaped to a great extent by the social, economic, and physical environments in which people live, and that social inequalities are associated with increased risk of many common mental disorders [52]”. For example dietary intake [53] and food insecurity have been shown to impact various mental health constructs [54].

Because of the small sample size and high drop-out rate seen with the 7-day post-intervention questionnaires received (inherent to the nature of mailed surveys [55], the study is insufficiently powered and limited in the ability to generalize findings. The high 7-day drop-out rate also resulted in the anxiety and communication results being mainly reflective of outpatients. The findings on anxiety, stress, pain, and communication can only suggest the possibility of a positive therapeutic effect.

Pain was only measured in inpatients, and at the time we designed the study, our thinking was that inpatients included those actively undergoing chemotherapy, radiation, and/or surgical treatment and would be those who could have significant amounts of physical pain. We recognize that outpatients and support group participants could also experience pain. Additionally, as a pilot study, this study did not have a control group receiving alternative treatment(s) to rule out a placebo effect; therefore, we cannot rule out participant expectancy of the intervention to work, leading to a placebo effect. We suggest that future research utilize a more rigorous study design such as a randomized control study, recruiting larger sample sizes and controlling groups to compare the effects of different mindful stress-reduction interventions. All the responses were self-reported and intrinsically subject to response bias. Future research could improve on this by exploring the physiologic effects of these interventions as well as examining whether the positive effects of the intervention translate into enhanced patient care. Lastly, the Option Dyadic Scale for Shared Decision-Making is not a formally validated and reliable instrument. To our knowledge, no reliability or validity testing has been completed in our target clinical population; therefore, we cannot reliably determine true interdependence between physician and patient responses [38].

While our results demonstrate the feasibility of such an intervention in both the inpatient and outpatient setting, it should be noted that our study was fully funded by a grant. Groups looking to implement such an intervention should therefore consider the cost implications.

Physicians, administrators, and organizations must be cognizant of the detrimental relationship stress and anxiety can have on patients and their treatment outcomes. Hospitalized patients or outpatients may be contending with fear of the unknown, loss of control, or outcomes associated with their treatment, including the possibility of death. These pervasive psychological factors may impede how patients respond or adhere to treatment, communicate with their providers and caregivers, and how they perceive the future.

## 5. Conclusions

This pilot study showed that the mindful stress-reduction interventions we implemented alleviated anxiety, stress, and perceived pain in participants in the short-term and was feasible in outpatient and acute care settings. The impact these interventions have on longer-term and downstream outcomes is still up for debate. Further studies of mindful stress-reduction interventions in selective populations with control groups for analysis, would provide additional information involving application success and opportunities for improvement.

## Figures and Tables

**Table 1:** Study questionnaires given by time point and treatment group.

	Study Time Point			
	Immediate Pre-Intervention	Immediate Post-Intervention	Immediate Post-Office Visit	7-Day Post-Intervention
Treatment Group	Instruments Administered			
Cancer inpatients	Perceived Stress, Anxiety, Communication, Pain	Perceived Stress, Pain	n/a	Anxiety, Communication
Cancer outpatients	Perceived Stress, Anxiety, Communication	Perceived Stress	n/a	Anxiety, Communication
Support group participants	Perceived Stress, Anxiety, Communication	Perceived Stress	Perceived Stress, Dyadic OPTION	Anxiety, Communication

OPTION (observing patient involvement).

**Table 2:** Participant Demographics (*n* = 59).

Variable	*n* (%)	Mean (SD)	Median (IQR)
Source			
Inpatient	23 (39.0)		
Outpatient	20 (33.9)		
Support Group Participants	16 (27.1)		
Age		60.6 (14.6)	66.5 (71.0–51.0)
25–44	8 (13.6)		
45–64	17 (28.9)		
65+	29 (49.1)		
Gender			
Female	34 (60.7)		
Male	22 (39.3)		
Race			
Black or African American	17 (30.4)		
White	33 (58.9)		
Other	6 (10.7)		
Ethnicity			
Mexican, Mexican American, or Chicano/a	2 (3.6)		
Not Hispanic, Latino/a, or Spanish origin	45 (81.8)		
Other Hispanic, Latino/a, or Spanish origin	6 (10.9)		
Unknown	2 (3.6)		
Diagnosis			
Breast Cancer	14 (23.7)		
Liver Cancer	4 (6.8)		
Pancreas Cancer	18 (30.5)		
Unknown/Other	23 (39.0)		

**Table 3:** Pre- and Post-Intervention Perceived Stress Levels.

Perceived Stress Level	Pre-Intervention *n* (%)	Post-Intervention *n* (%)	Post MD Visit * n (%)
1 = Feeling great!	4 (7.0)	18 (33.3)	2 (18.2)
2 = Feeling good	11 (19.3)	26 (48.1)	7 (63.6)
3 = A little stressed	23 (40.3)	10 (18.5)	2 (18.2)
4 = Definitely stressed	14 (24.6)	0 (0.0)	0 (0.0)
5 = Stressed out!	5 (8.8)	0 (0.0)	0 (0.0)

* Only outpatients who attended an appointment with their physician were asked what their perceived stress level was following that appointment.

**Table 4:** Pre- and post-intervention anxiety and communication scale raw scores, and pain VAS levels.

Study Instrument	Pre-Intervention	Post-7 Day Intervention	Paired *t*-test
Anxiety Scale Raw Score			
*n*	55	16	t(14) = 3.18, ***p* = 0.0067**
Mean (SD)	56.6 (17.7)	41.2 (11.9)	
Median (IQR)	54.0 (66.0–41.0)	43.5 (50.0–31.5)	
Communication Scale Raw Score			
*n*	55	16	t(14) = −2.82, ***p* = 0.0135**
Mean (SD)	81.7 (17.9)	90.6 (14.0)	
Median (IQR)	85.0 (95.0–70.0)	95.0 (100.0–87.5)	
	**Pre-Intervention**	**Post-Intervention**	
Pain VAS *			
*n*	21	21	t(20) = −12.96, ***p* < 0.0001**
Mean (SD)	7.0 (2.5)	2.8 (2.8)	
Median (IQR)	7.0 (8.0–6.0)	2.0 (5.0–1.0)	

Statistically significant differences (*p* < 0.05) are bolded. * Only inpatients were asked about their pain score using the VAS.

**Table 5:** Association between Pre-Intervention Anxiety and Stress.

Pre-Intervention Perceived Stress Levels	Average Pre-Intervention Anxiety Raw Scores	ANOVA
Feeling great!	50.3 ± 13.0	*p* = **0.0195**
Feeling good	48.6 ± 14.4	
A little stressed	52.8 ± 16.5	
Definitely stressed	64.8 ± 16.3	
Stressed out!	73.6 ± 21.3	

Statistically significant differences (*p* < 0.05) are bolded.

**Table 6:** Group Correlations.

Study Time Period by Instrument Used		Pre-Intervention Anxiety	Post-7-Day Intervention Anxiety	Pre-Intervention Communication	Post-7- Day Intervention Communication	Pre-Intervention VAS	Post-Intervention VAS
**Pre-Intervention Anxiety**	Correlation	1		**−0.49428**	−0.26405	0.32497	0.26735
	*p*	-		0.0002	0.3416	0.1506	0.2414
**Post-7-day Intervention Anxiety**	Correlation		1	−0.28904	**−0.53468**	−0.27735	0.93677
	*p*		-	0.2961	**0.0329**	0.8211	0.2276
**Pre-Intervention Communication**	Correlation	−0.49428	−0.28904	1		0.17066	−0.41255
	*p*	0.0002	0.2961	-		0.8908	0.0631
**Post-7-day Intervention Communication**	Correlation	−0.26405	**−0.53468**		1	0.17066	−0.89290
	*p*	0.3416	**0.0329**		-	0.8908	0.2973
**Pre-Intervention VAS**	Correlation	0.32497	−0.27735	−0.10710	0.17066	1	
	*p*	0.1506	0.8211	0.6440	0.8908	-	
**Post-Intervention VAS**	Correlation	0.26735	0.93677	−0.41255	−0.89290		
	*p*	0.2414	0.2276	0.0631	0.2973		

Statistically significant differences (*p* < 0.05) are bolded.

**Table 7:** Dyadic Shared Decision-Making OPTION Scale.

Item	Question	Response	Patient *n* = 11	Physician *n* = 8
**Item 1**	A health problem was identified, where it was made clear that a decision was needed	Strongly agree/agree	10	8
		Disagree/strongly disagree	1	0
**Item 2**	More than way to manage the health problem was described	Strongly agree/agree	10	8
		Disagree/strongly disagree	1	0
**Item 3**	Different sources of information (e.g., leaflets, websites, contact with other people) to help make the decisions were offered	Strongly agree/agree	9	8
		Disagree/strongly disagree	2	0
**Item 4**	Different options (including the possibility of doing nothing) were discussed	Strongly agree/agree	9	8
		Disagree/strongly disagree	2	0
**Item 5**	The advantages, disadvantages and possible outcomes of options were discussed	Strongly agree/agree	10	8
		Disagree/strongly disagree	1	0
**Item 6**	Ideas or expectations about managing the health problem were discussed	Strongly agree/agree	10	8
		Disagree/strongly disagree	1	0
**Item 7**	Concerns or worries about managing the health problem were discussed	Strongly agree/agree	8	8
		Disagree/strongly disagree	3	0
**Item 8**	It was made sure that information had been understood	Strongly agree/agree	11	8
		Disagree/strongly disagree	0	0
**Item 9**	There were opportunities to ask questions	Strongly agree/agree	10	8
		Disagree/strongly disagree	1	0
**Item 10**	The preference to take part in the decision (or not) was respected	Strongly agree/agree	11	8
		Disagree/strongly disagree	0	0
**Item 11**	During the consultation, a decision was made; or there was an agreement to postpone making the decision	Strongly agree/agree	10	8
		Disagree/strongly disagree	1	0
**Item 12**	The possibility of coming back to the decision was discussed	Strongly agree/agree	9	8
		Disagree/strongly disagree	2	0

## Data Availability

The data that support the findings of this study are available from Methodist Health System but restrictions apply to the availability of these data, which were used under license for the current study, and so are not publicly available. Data are however available from the authors upon reasonable request and with permission of Methodist Health System.

## Abbreviations

ANOVA	Analysis of variance
CONSORT	Consolidated Standards of Reporting Trials
MBSR	Mindfulness-based stress reduction
MDMC	Methodist Dallas Medical Center
PYTI-C	Professional Yoga Therapy Institute certified
SAS	Statistical Analysis System
VAS	Visual analog scale

## References

[1] Kochanek K.D., Xu J.Q., Arias E. (2020). Mortality in the United States, 2019. NCHS Data Brief, no 395.

[2] (2020). American Cancer Society: Cancer Facts and Figures 2020.

[3] (2020). Types of Cancer Treatment.

[4] Adler N.E., Page A.E.K. (2008). The Psychosocial Needs of Cancer Patients. Cancer Care for the Whole Patient: Meeting Psychosocial Health Needs.

[5] Hickman R.L., Douglas S.L. (2010). Impact of chronic critical illness on the psychological outcomes of family members. Aacn Adv Crit Care.

[6] Vivian E., Oduor H., Lundberg L., Vo A., Mantry P.S. (2019). A Cross-Sectional Study of Stress and the Perceived Style of Decision-Making in Clinicians and Patients With Cancer. Health Serv. Res. Manag. Epidemiol..

[7] Kotrotsiou E., Theodosopoulou E., Papathanasiou I., Gr D., Raftopoulos V., E K. (2001). How do patients experience stress caused by hospitalization and how do nurses perceive this stress experienced by patients. A comparative study. Icus Nurs. Web J..

[8] Musazzi L., Tornese P., Sala N., Popoli M. (2017). Acute or Chronic? A Stressful Question. Trends Neurosci..

[9] Herbert T.B., Cohen S. (1993). Stress and immunity in humans: A meta-analytic review. Psychosom. Med..

[10] Cohen S., Miller G.E., Rabin B.S. (2001). Psychological stress and antibody response to immunization: A critical review of the human literature. Psychosom. Med..

[11] Vivian E., Manhas A., Lee A., Deluna C., Oduor H., Vo A., Davis A., Worral S., Mantry P. Evaluation of Oncology Patient Experiences and Preferences for Shared Decision Making and Patient-Centered Care. Proceedings of the Institute for Health Improvement National Forum.

[12] Hofmann S.G., Sawyer A.T., Witt A.A., Oh D. (2010). The effect of mindfulness-based therapy on anxiety and depression: A meta-analytic review. J. Consult. Clin. Psychol..

[13] Mackenzie M.J., Carlson L.E., Munoz M., Speca M. (2007). A qualitative study of self-perceived effects of mindfulness-based stress reduction (MBSR) in a psychosocial oncology setting. Stress Health.

[14] Farb N.A., Anderson A.K., Mayberg H., Bean J., McKeon D., Segal Z.V. (2010). Minding one’s emotions: Mindfulness training alters the neural expression of sadness. Emotion.

[15] Hargus E., Crane C., Barnhofer T., Williams J.M.G. (2010). Effects of mindfulness on meta-awareness and specificity of describing prodromal symptoms in suicidal depression. Emotion.

[16] Jha A.P., Stanley E.A., Kiyonaga A., Wong L., Gelfand L. (2010). Examining the protective effects of mindfulness training on working memory capacity and affective experience. Emotion.

[17] Aherne D., Farrant K., Hickey L., Hickey E., McGrath L., McGrath D. (2016). Mindfulness based stress reduction for medical students: Optimising student satisfaction and engagement. Bmc Med Educ..

[18] Moore A., Malinowski P. (2009). Meditation, mindfulness and cognitive flexibility. Conscious. Cogn..

[19] Verweij H., van Ravesteijn H., van Hooff M.L.M., Lagro-Janssen A.L.M., Speckens A.E.M. (2018). Mindfulness-Based Stress Reduction for Residents: A Randomized Controlled Trial. J. Gen. Intern. Med..

[20] Will A., Rancea M., Monsef I., Wöckel A., Engert A., Skoetz N. (2015). Mindfulness-based stress reduction for women diagnosed with breast cancer. Cochrane Database Syst. Rev..

[21] (2019). 2019 Cancer Patient Experience Survey Results.

[22] Williams A. (1998). Therapeutic landscapes in holistic medicine. Soc Sci Med.

[23] Verderber S. (1986). Dimensions Ofperson-Window Transactionsin the Hospital Environment. Environ Behav.

[24] Garner G. (2016). Medical Therapeutic Yoga: Biopsychosocial Rehabilitation and Wellness Care.

[25] Gallego J., Nsegbe E., Durand E. (2001). Learning in respiratory control. Behav. Modif..

[26] Coulter D.H. (2010). Anatomy of Hatha Yoga: A Manual for Students, Teachers, and Practitioners.

[27] Porges S.W. (2001). The polyvagal theory: Phylogenetic substrates of a social nervous system. Int. J. Psychophysiol. Off. J. Int. Organ. Psychophysiol..

[28] Brown R.P., Gerbarg P.L. (2012). The Healing Power of the Breath: Simple Techniques to Reduce Stress and Anxiety, Enhance Concentration, and Balance Your Emotions.

[29] Vaschillo E.G., Vaschillo B., Lehrer P.M. (2006). Characteristics of resonance in heart rate variability stimulated by biofeedback. Appl. Psychophysiol. Biofeedback.

[30] Mellin L. (2011). Emotional Brain Training for Treating Obesity, Eating Disorders, and Stress. Scan’s Pulse.

[31] Vivian E., Oduor H., Arceneaux S.R., Flores J.A., Vo A., Madden B.M. (2019). A Cross-Sectional Study of Perceived Stress, Mindfulness, Emotional Self-Regulation, and Self-Care Habits in Registered Nurses at a Tertiary Care Medical Center. Sage Open Nurs..

[32] Cella D., Lai J.S., Nowinski C.J., Victorson D., Peterman A., Miller D., Bethoux F., Heinemann A., Rubin S., Cavazos J.E. (2012). Neuro-QOL: Brief measures of health-related quality of life for clinical research in neurology. Neurology.

[33] Gershon R.C., Lai J.S., Bode R., Choi S., Moy C., Bleck T., Miller D., Peterman A., Cella D. (2012). Neuro-QOL: Quality of life item banks for adults with neurological disorders: Item development and calibrations based upon clinical and general population testing. Qual. Life Res..

[34] Neuro QoL-Validation. Healthmeasures.net.

[35] Cella D. (2015). User Manual for the Quality of Life in Neurological Disorders (Neuro-QoL) Measures.

[36] McCormack H.M., Horne D.J., Sheather S. (1988). Clinical applications of visual analogue scales: A critical review. Psychol. Med..

[37] Knop C., Oeser M., Bastian L., Lange U., Zdichavsky M., Blauth M. (2001). [Development and validation of the Visual Analogue Scale (VAS) Spine Score]. Unfallchirurg.

[38] Melbourne E., Sinclair K., Durand M.A., Legare F., Elwyn G. (2010). Developing a dyadic OPTION scale to measure perceptions of shared decision making. Patient Educ. Couns..

[39] Nicolai J., Moshagen M., Eich W., Bieber C. (2012). The OPTION scale for the assessment of shared decision making (SDM): Methodological issues. Z. Fur EvidenzFortbild. Und Qual. Im Gesundh..

[40] Hoffman C.J., Ersser S.J., Hopkinson J.B., Nicholls P.G., Harrington J.E., Thomas P.W. (2012). Effectiveness of mindfulness-based stress reduction in mood, breast- and endocrine-related quality of life, and well-being in stage 0 to III breast cancer: A randomized, controlled trial. J. Clin. Oncol. Off. J. Am. Soc. Clin. Oncol..

[41] Branstrom R., Kvillemo P., Brandberg Y., Moskowitz J.T. (2010). Self-report mindfulness as a mediator of psychological well-being in a stress reduction intervention for cancer patients--a randomized study. Ann. Behav. Med. A Publ. Soc. Behav. Med..

[42] Vollestad J., Sivertsen B., Nielsen G.H. (2011). Mindfulness-based stress reduction for patients with anxiety disorders: Evaluation in a randomized controlled trial. Behav. Res. Ther..

[43] Goldin P.R., Gross J.J. (2010). Effects of mindfulness-based stress reduction (MBSR) on emotion regulation in social anxiety disorder. Emotion.

[44] Bahrami F., Yousefi N. (2011). Females are more anxious than males: A metacognitive perspective. Iran J. Psychiatry Behav. Sci..

[45] Lindemann C. (1996). Handbook of the Treatment of the Anxiety Disorders.

[46] McLean C.P., Asnaani A., Litz B.T., Hofmann S.G. (2011). Gender differences in anxiety disorders: Prevalence, course of illness, comorbidity and burden of illness. J. Psychiatr Res..

[47] Barre V.P., Padmaja G., Saxena R.K., Rana S. (2015). Impact of medical intervention on stress and quality of life in patients with cancer. Indian J. Palliat. Care.

[48] Bultz B.D., Carlson L.E. (2006). Emotional distress: The sixth vital sign--future directions in cancer care. Psycho Oncol..

[49] Li Q., Lin Y., Xu Y., Zhou H. (2018). The impact of depression and anxiety on quality of life in Chinese cancer patient-family caregiver dyads, a cross-sectional study. Health Qual. Life Outcomes.

[50] Northouse L.L., Mood D., Templin T., Mellon S., George T. (2000). Couples’ patterns of adjustment to colon cancer. Soc. Sci. Med..

[51] Hagedoorn M., Sanderman R., Bolks H.N., Tuinstra J., Coyne J.C. (2008). Distress in couples coping with cancer: A meta-analysis and critical review of role and gender effects. Psychol. Bull.

[52] (2014). Social Determinants of Mental Health.

[53] Głąbska D., Guzek D., Groele B., Gutkowska K. (2020). Fruit and Vegetable Intake and Mental Health in Adults: A Systematic Review. Nutrients.

[54] Pourmotabbed A., Moradi S., Babaei A., Ghavami A., Mohammadi H., Jalili C., Symonds M.E., Miraghajani M. (2020). Food insecurity and mental health: A systematic review and meta-analysis. Public Health Nutr..

[55] (2020). Mail Surveys.

